# The Treatment of Psychoses by Opium

**Published:** 1889-10

**Authors:** 


### THE TREATMENT OF PSYCHOSES BY OPIUM.

In a a recent number of the *Therapeutische Monatsheft* (Berlin), Dr. Ziehen, of Jena, publishes his results of the administration of opium in various psychoses.

In the past two years, ninety-seven patients have undergone treatment; forty-three cases of melancholia, four of mania, fifty of paranoia. He administered the opium three times daily, beginning with doses of one-half grain, and gradually increasing until in some cases sixteen to twenty-four grains were given daily. This was kept up for weeks at a time, according to the case under treatment. The opium was seldom given hypodermically, generally per os, and in the form of pure opium.

The results obtained were highly satisfactory, especially in the cases of melancholia. Of thirty-nine patients afflicted with melancholia, thirty-one recovered, or seventy-nine per cent. In those cases in which patients were troubled much with illusions, the results were not so favorable. In eight such cases, two only recovered. Particularly in senile melancholia did the opium render excellent service, also in melancholia hallucinatoria, and in those forms with fixed ideas. In melancholia "passiva" and "stupida," opium alone proved of little value, but combined with camphor (three-fourths to one-half grain daily), good results were obtained.

In mania, no benefit was derived from this treatment; the bromides and hyoscin were preferable.

In paranoia, the two forms, with hallucinations, and without hallucinations, are to be kept distinct. In the latter form (paranoia siniplex), opium was contra-indicated. In "paranoia hallucinatoria," where cerebral exhaustion is the important factor, small doses, of three to nine grains, proved beneficial as long as hallucinations were present. In twenty-eight such cases, Ziehen reported twenty-four recoveries, or eighty-six per cent.

Ziehen advises this treatment to be given as early as possible in the course of the disease, and may be carried on with perfect safety in private practice.[1] W. C. K.

1. This mode of treatment has not only produced good results in the hands of Dr. Ziehen, but several German observers recommend it very highly. Professor Mendel, in Berlin, has adopted it in his private hospital for the treatment of psychoses, and has been unusually successful. Particularly in "melancholia hallucinatoria" has the opium treatment rendered him excellent service, a large percentage terminating in recovery. The success which has attended two such psychiaters, as Ziehen and Mendel, should stimulate observers in this country to give this procedure a good and thorough trial. Bigoted motives should not deter one from doing his honest duty, even though it be the feeding of insane with opium.—W. C. K.

